# Analysis on Fungal Diversity in Rhizosphere Soil of Continuous Cropping Potato Subjected to Different Furrow-Ridge Mulching Managements

**DOI:** 10.3389/fmicb.2017.00845

**Published:** 2017-05-10

**Authors:** Shuhao Qin, Stephen Yeboah, Xuexue Xu, Yuhui Liu, Bin Yu

**Affiliations:** ^1^College of Horticulture, Gansu Agricultural UniversityLanzhou, China; ^2^Gansu Key Laboratory of Crop Genetic and Germplasm Enhancement, Gansu Agricultural UniversityLanzhou, China; ^3^CSIR-Crops Research InstituteKumasi, Ghana

**Keywords:** continuous cropping soil, fungal diversity, furrow-ridge mulching (FRM), potato

## Abstract

Knowledge about fungi diversity following different planting patterns could improve our understanding of soil processes and thus help us to develop sustainable management strategies. The objective of this study was to determine the impact of different furrow-ridge mulching techniques on fungal diversity in rhizosphere soil under continuous cropping system. The investigated treatments were: flat plot without mulch (CK); flat plot with mulch (T1); on-ridge planting with full mulch (T2); on-furrow planting with full mulch (T3); on-ridge planting with half mulch (T4); and on-furrow planting with half mulch (T5). NGS (Illumina) methods and ITS1 sequences were used in monitoring fungi diversity of the potato rhizosphere soil. The fungi diversity in the rhizosphere soil was ranked in the order T5 > T2 > T4 > T1 > CK at the early growth stage and T2 > T3 > T1 > T4 > CK at the late growth stage of potato. The fungal communities found in the rhizosphere soil were Ascomycota, Zygomycota, Basidiomycota, Chytridiomycota, and other unidentified fungal communities. Among the fungal community in the rhizosphere soil, Ascomycota was found to be dominant fungi population, with the highest percentage (89%) in the T5 soil whereas the T2 soils had the lowest percentage (67%). The *Fusarium* abundance in fully-mulched treated soils was higher than in half-mulched treated soil. The dominant genus in the T4 soil was *Mortierella*, whereas lower populations (1–2%) of *Scutellinia*, *Cryphonectria*, *Acremonium*, and *Alternaria* were found in that treatment. Among the eumycetes, the dominant fungal class in all treated soils was the Sordariomycetes, which ranged from 57 to 85% in T2 and T5 soils, respectively. The *Fusarium* percentages in half-mulched treated soils (T4 and T5) were 55 and 28% lower than that of complete mulched treated soils (T2 and T3), respectively. The cluster analysis results showed that, CK, T4, and T5 treated soils and T1, T2, and T3 treated soils had similarities in microbial compositions, respectively. Potato tuber yield was greater under the on-ridge planting with full mulch (T2) treated soil, followed by on-ridge planting with half-mulch (T4) treated soil. The rhizosphere soil under the on-ridge planting with full-mulch (T2) soil had the highest fungal diversity, suggesting that this management was the best environment for fungi, whereas the on-ridge planting with half-mulch (T4) soil had the minimum abundance of *Fusarium.*

## Introduction

The problems of potato fungal diseases on plants were probably observed even while humans were hunter-gatherers (Agrios, 2005). However, it is generally agreed that during this period when dependence was on natural population of plants, the extent of the damage due to diseases was variable and usually localized (Scheffer, 1997). When agriculture began and certain food crops were planted the incident of plant diseases increased and in some years caused famines or at least greatly reduced the available amount of food (Agrios, 2005). Potato (*Solanum tuberosum* L.) is the world’s most important non-grain food crop, is central to global food security and regarded as an advantageous crop with better economic benefit compared with other crops in western area in the Loess Plateau of China (Wang et al., 2008; Qin et al., 2014). However, potato was mostly planted using non-mulching planting management in the region in the past several years. Consequently the yield of potato was low, and its profitability and water use efficiency (WUE) were also low (Qin et al., 2013). In recent years, the tuber yield and WUE of potato has been greatly improved with the use of ridge and film mulching technology (Zhao et al., 2012; Qin et al., 2014). The consistent increased in potato planting area over the years has resulted in continuous potato cropping. The growth and development of potato is greatly inhibited because of extensive potato continuous cropping in this region (Wang et al., 2015), and the occurrence of diseases caused by soil-borne pathogens (Alvey et al., 2003; Aparicio and Costa, 2007). It is well established that microbes affect absorption and transformation of nutrients in soil (Zak et al., 1993; Rogers et al., 1994) and the imbalance of microbial population composition can decrease soil quality and crop yield (Pampolino et al., 2006; Zhou et al., 2008; Fu et al., 2009; Ma et al., 2010). Continuous cropping, single nutrient consumption and declined of soil fertility reduced microbial composition in soil and promote the growth of harmful soil microbes (Lu et al., 2010). Under the condition of continuous cropping, some potato diseases, such as early blight, late blight, blight, dry rot, and black scurf caused by some fungal, has a higher occurrence rate in the research region. The main toxins of these diseases produced by *Fusarium solani*, *Fusarium semitectum*, *Fusarium moniliforme*, *Fusarium oxysporum*, *Rhizoctonia solani*, *Phytophthora infestans*, *Alternaria* and so on in North-west of China (Peng and Zhu, 2008). Methods to determine microbial community composition, such as microbial dilution methods, Biolog identification system and biomarkers often underestimate soil microbial diversity (Klingler et al., 1992) whereas sequencing using Illumina MiSeq method analysis can partially avoid this disadvantage (Williams et al., 2014; Schirmer et al., 2015). MiSeq’s high-throughput sequencing platform not only realizes simultaneous sequencing of several samples, but it is also rapid and accurate. For this reason, this platform is widely applied to study microbial diversity (Konstantinidis and Tiedje, 2005; Sogin et al., 2006; Lauber et al., 2009).

Existing reports on the effect of potato continuous cropping on rhizosphere soil microbial community composition d is mainly on traditional planting methods couple with non-mulching practices (Li and Guo, 2014). The influence of potato continuous cropping with different ridge-furrow mulching planting managements on soil microbial diversity are limited to know on arid land. Therefore, we conducted this experiment to explore the diversity of fungal community of rhizosphere soil as affected by 2- to 3-year potato continuous cropping in furrow-ridge mulching (FRM) by using high-throughput sequencing method.

## Materials and Methods

### Description of the Experimental Site

The field experiment was conducted in 2013 and 2014 at the Experimental Station (35°33′N, 104°35′E, elevation 1874 m a.s.l.) of Rain-fed Agricultural Research Institute of Gansu Agricultural University, at Dingxi, Northwest China. Fungal diversity was determined in the Gansu Key Laboratory of Crop Genetic and Germplasm Enhancement of Gansu Agricultural University. The experimental site had a Huangmian soil with deep soil layer, high water-storing capacity, wilting percentage at 7.3% and mid-level soil fertility, in the Chinese soil taxonomy (Chinese Soil Taxonomy Cooperative Research Group, 1995); It is a Calcaric Cambisols in the FAO classification (FAO, 1990) and it is a typical soil in the Loess Plateau. The average long-term annual rainfall at Dingxi is 402 mm (1970–2014). Daily maximum temperatures can reach 38°C in July, while minimum temperatures can drop to -22°C in January. The annual average radiation is 5929 MJ/m^2^, and sunshine 2477 h per year.

### Experimental Design

**FIGURE 1:**
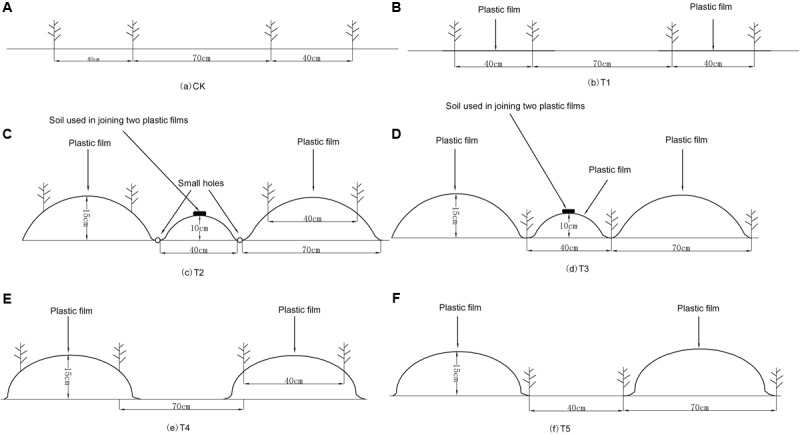
**The schematic diagrams of different treatments in both experiment years. (A)** CK, non-film mulched flat plot; **(B)** T1, half-mulched flat plot (70 cm) alternate with strips of bare land (40 cm) without ridges. Potato planted in two rows in the mulched plot and spaced at 40 cm; **(C)** T2, fully mulched ridge cropping (70 cm) alternate with narrow ridges (40 cm) and ridge cropping. Potato planted in two rows in the mulched ridges and spaced at 40 cm; **(D)** T3, fully mulched furrow cropping (70 cm) with narrow ridges (40 cm). This was similar to T2 with the exception of planting at the bottom of the furrows (two furrows); **(E)** T4, half-mulched ridge cropping (70 cm) alternate with bare land (40 cm) that had no ridges and mulching. Potato were planted in two rows in the mulched ridges plots and spaced at 40 cm. **(F)** T5, half-mulched furrow cropping (70 cm) alternate with bare land (40 cm) that had no ridges and mulching. Two rows of potato were planted in the non-mulched plots and spaced at 40 cm.

**FIGURE 2:**
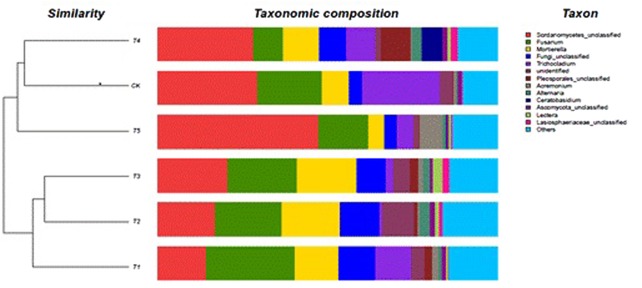
**Fungal genera in the different soils**.

**FIGURE 3:**
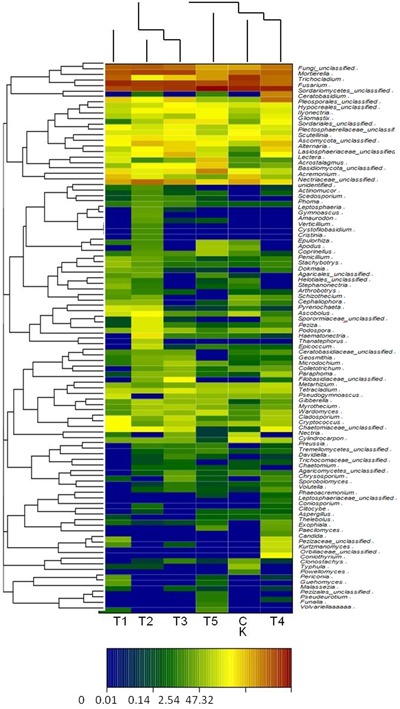
**The heatmap of soil fungal genera in the different soils.** A basis of the vertical and horizontal clustering present an OTU (species) in the Figure. The similarities and differences of community composition from different samples under the classification level were reflected by color gradient and similar degree.

**FIGURE 4:**
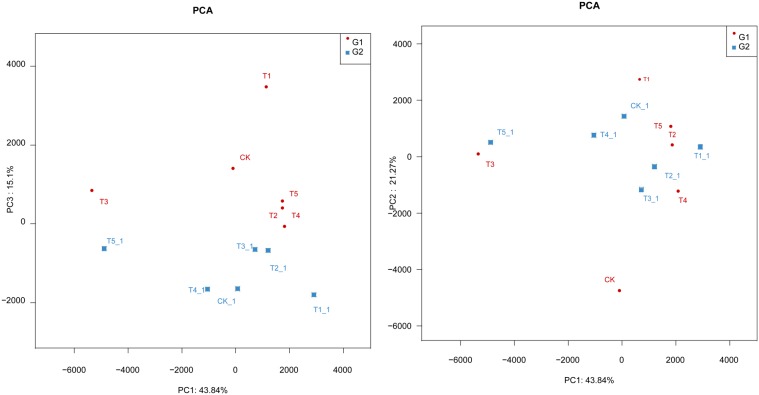
**Principal component analysis (PCA) of soil fungal community in the different soils; G1: 08-July, G2: 15-September**.

**FIGURE 5:**
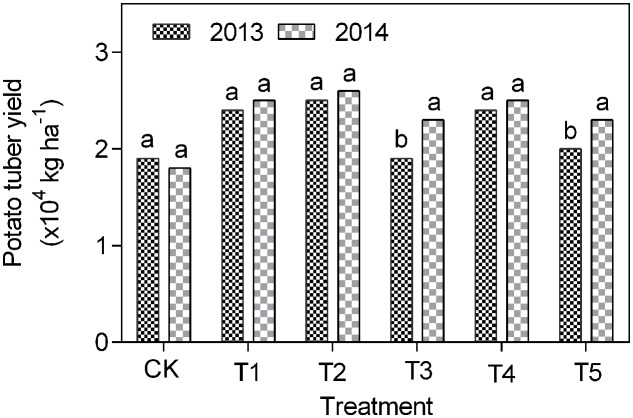
**Potato tuber yield in different treatments under continuous cropping**.

This experiment was conducted in a potato continuous cropping field. The study reported here was conducted in 2013 and 2014 which were the second and the third continuous cropping years, respectively. The following six treatments were used in a randomized, complete block design with three replicates in each year: (1) a flat plot without mulch (CK) (**Figure 1A**); (2) alternating strip mulched with plastic film (70 cm) with strip of bare land (40 cm) with no ridges (designated as T1). Two rows of potato were planted in the mulched plot and spaced at 40 cm (**Figure 1B**); (3) alternating fully-mulched wide ridges (70 cm) with narrow ridges (40 cm) and ridge planting (designated as T2). All the ridges and furrows were mulched with plastic film, an innovative water saving technology in arid areas. Two rows of potato were planted in the ridges with dibblers, spaced at 70 and 40 cm with wide and narrow rows, respectively (**Figure 1C**); (4) alternating fully-mulched wide ridges (70 cm) with narrow ridges (40 cm) and furrow planting (designated as T3). The treatment application was the same as T2 with the exception of planting in the furrows (two rows) with dibblers (**Figure 1D**); (5) ridges mulched with plastic film (70 cm) were alternated with bare land (40 cm) that had no ridges and no mulches (designated as T4). Two rows of potato were planted in the mulched ridges plots and spaced at 40 cm (**Figure 1E**); (6) ridges mulched with plastic film (70 cm) were alternated with bare land (40 cm) that had no ridges and no mulches (designated as T5). Two rows of potato were planted in the non-mulched plots and spaced at 40 cm (**Figure 1F**).

The black plastic film used in all treatments was 0.01 mm thick. The ridges were formed and the plastic sheets were placed with a ridging-film machine on 15 April 2013 and 17 April 2014. Soil was heaped on the film in bands every 3 m to prevent wind from shifting the plastic sheeting. Small holes were made in the film of furrow every 30 cm to permit rainfall infiltration. The potato cultivar “Xindaping” was planted at the density of 45460 plants ha^-1^ on April 25, 2013 and April 26, 2014 using a dibbler. Plots were 16 m × 11 m in size. There was a buffering area between plots (100 cm) and between blocks (120 cm). All potatoes were harvested on October 1, 2013 and October 2, 2014. Nitrogen, phosphorus, and potassium were applied by broadcasting at a rate of 96 kg N⋅ha^-1^ (urea), 84 kg P_2_O_5_⋅ha^-1^ (calcium superphosphate), and 120 kg K_2_O⋅ha^-1^ (potassium sulfate), respectively. The previous crop prior to setting up the experiment was peas.

### Soil Samples Collection

The soil samples were collected using a dry-sterile brush to brushing the surface soil of potato root into sterile ziplock bags after removing large particles, broken roots and stone which were then transferred to the laboratory for analysis. The soil was sampled at six points in each plot, and then mixed into one sample. The samples were put in an ice box and taken to the laboratory, and stored at -80°C for DNA extraction of soil microbes. The other half soil samples were air-dried and sieved (<1 mm) for determination of physical and chemical properties and enzyme activity from soil.

### Soil DNA Extraction

DNA was extracted from soil (0.25 g) by the Power Soil DNA Isolation kit (MOBIO Laboratories, Inc., US). The quality of extracted DNA was checked by Gold View staining after 1% agarose gel electrophoresis (AGE). Amplification of fungal ITS region was done using ABI GeneAmp 9700 PCR instrument with 1737F 5′-GGAAGTAAAAGTCGTAACAAGG-3′ and 2043R 5′-GCTGCGTTCTTCATCGATGC-3′ primers. The PCR reaction was carried out in 20 μl, containing 4 μl 5 (Diluted multiples) × FastPfu Buffer, 2 μl 2.5 mM dNTPs, 0.8 μl Forward Primer (5 μM), 0.8 μl Reverse Primer (5 μM), 0.4 μl FastPfu Polymerase, 10 ng Template DNA and dd H_2_O added up to volume. Fluorescence of PCR products was determined by recycling PCR product with AxyPrepDNA gel recovery kit (Axygen Inc., Union City, CA, USA) and amplicons were eluted using Tris-HCL and thereby quantified. High-throughput sequencing was performed using Illumina MiSeq platforms at the Gansu Key Laboratory of Crop Genetic and Germplasm Enhancement. The NGS pipeline was presented in **Figure 2**.

### Soil Enzyme Activity

Peroxidase activity was determined using KMnO_4_ titration method, urease activity using phenol sodium hypochlorite colorimetric method, protease activity using *N*-benzoyl-L-Arginine ethylester (Zhou, 1987) and alkaline phosphomonoesterase activity was measured as reported by Colvan et al. (2001) and Nannipieri et al. (2011).

### Potato Tuber Yield

The tuber yield per hectare (kg/ha) was obtained by determining the tuber yield of each plot in each year at harvest

### Statistical Analysis

Both statistical and principal component analysis (PCA) were carried out in the different treated soils Using SPSS19.0 and CANOCO for windows 4.5. Statistical analyses were performed with analysis of variance (ANOVA) at *P* < 0.05 using Statistical Package for the Sciences 22.0 (IBM Corporation, Chicago, IL, USA).

## Results

### Soil Fungal Diversity Indices and Richness

In this study, the Ribosomal Database Project (RDP) classifier was used for the taxonomic analysis on 97% similarity level of OTU representative sequences. The diversity indices mainly include: richness index *Chao*, diversity index *Shannon*, and sequencing depth and *Coverage* (**Table 1**). More than 99% of coverage indices indicated that the sequencing results at a similarity level of 0.03 can reflect the true fungal diversity in tested samples. At the early growth stage of potato (08-June), the fungal diversity in T1, T2, T4, and T5 soils was higher than that of CK soil by 16.4, 26.9, 17.8, and 28.4%, respectively. At the later growth stage of potato (15-September), the fungal diversity in T1, T2, T3, and T4 soils was higher than that of CK soil by 24.5, 40.7, 29.6, and 19.5%, respectively. However, T3 and T5 soils were significantly lower in fungi diversity than CK soil at the early and late growth stages, respectively. Two fully mulched treated (T2 and T3) soils had the highest fungal diversity at the late growth stage of potato, and the lowest value in T5 soil. FRM treatments also increased the fungal richness compared to CK soil. The OTU richness values could be ranked as T2 > T5 > T3 > T1 > CK > T4 at the early growth stage, and T2 > T1 > T3 > T4 > T5 > CK at the late growth stage. There was significant difference between the T2 and CK soils (**Table 1**).

**Table 1:** Soil fungal diversity and richness of different furrow-ridge mulching planting patterns.

Treatment	08-July				15-September			
	OTU^#^	Chao Richness index^&^	Shannon index^$^	Coverage %^∗^	OTU^#^	Chao Richness index^&^	Shannon index^$^	Coverage %^∗^
CK	227	284ab	2.75d	99.74	215	253b	2.77e	99.67
T1	228	290ab	3.20c	99.79	238	295ab	3.45c	99.41
T2	260	310a	3.49a	99.75	283	309a	3.90a	99.80
T3	240	291ab	2.70e	99.76	261	291ab	3.59b	99.81
T4	219	241b	3.24b	99.83	249	290ab	3.31d	99.77
T5	275	309ab	3.53a	99.80	239	262ab	2.62f	99.79


*^#^Stands for OTU number, ^&^for OTU richness, ^$^for microbial diversity, and ^∗^for library coverage of samples. Different letters represent significant difference (P <0.05)*.

### Analysis of Soil Fungal Community Composition

The most abundant were *Mortierella, Trichocladium*, and *Fusarium*; with the latter being dominant in the CK, T1, T2, T3, and T5 soils (**Figure 2**). The *Fusarium* abundance could be ranked as: T1 > T3 > T2 > CK > T5 > T4 and has the highest (26%) in T1 soil and the lowest in the T4 (8.9%) soil, being 54% lower than that of CK soil. The *Fusarium* abundance in fully-mulched treated soils was richer than in half-mulched treated soil. The dominant genus in the T4 soil was *Mortierella*. In addition, *Scutellinia, Cryphonectria*, *Acremonium*, and *Alternaria* were also present but in a low percentage (1–2%). The six treated soils were clustered into two groups: the first group included T4 (half-mulched with ridge planting), T5 (half-mulched with furrow planting), and CK (non-mulched flat plot) soil. The other group included T1 (mulching on flat plot), T2 (fully-mulched with ridge planting), and T3 (fully-mulched with furrow planting) soils.

### Phylogeny of Soil Fungi

Diversity and richness of fungal communities of the six treated soils are shown as color gradients and similarity degrees in Heatmap (**Figure 3**). These fungal communities were classified into 4 phyla, 20 classes, 48 orders, and 156 genera. The continuous cropping soil mainly showed Ascomycota, Zygomycota, Basidiomycota, Chytridiomycota, and unclassified fungi; Ascomycota was the dominant (88.9%) in the T5 (half-mulched with furrow planting), followed by Zygomycota. Ascomycota in the T2 (fully-mulched with ridge planting) soil accounted for 67.2%. Zygomycota showed the highest percentage (17.5%) in the T3 soil, whereas the lowest percentage (4.6%) was in the T5 soil. In addition, Chytridiomycota were not identified in T1, T4, and T5 soils, and were present at low percentages (0.01–0.05%) in the other treated soils. Eumycetes included Agaricomycetes, Ascomycetes, Basidiomycotes, Chytridiomycetes, Dothideomycetes, Eurotiomycetes, Leotiomycetes, Orbiliomycetes, Sordariomycetes, and Zygomycotes, with Sordariomycetes being dominant in all treated soils, ranging from 56.8% in T2 soil to 84.5% in the T5 soil. Hypocreales, abundance could be ranked as T1 > T3 > T5 > T2 > CK > T4, ranging from 25.8% in the CK soil to 34.4% in the TI soil. The genera with higher richness were *Fusarium*, *Mortierella*, and *Trichocladium* (**Figure 3**).

### The Principal Component Analysis (PCA) of Soil Fungal Community

The first three (PC1, PC2, and PC3) components accounted for 44, 21, and 15% with a whole variance of 80.2% (**Figure 4**). The distribution of the data points between different treatments showed a large interval, indicating that composition of fungal community in the soils under different management varies. In addition, the PC3 showed higher influence at early growth than at late growth stages as shown by the main constituent 2 (PC2). The composition of the fungal community of CK, T4, and T5 soils was similar, and the same also occurred for the T1, T2, and T3 soils. Similar results were found by the clustering analysis, and this indicates that, the different planting management affects the composition of soil fungal community (**Figure 4**).

### Potato Tuber Yield

The potato tuber yield in 2013 was increased under T1 to T5 treatments by 4–19% compared to CK plots (**Figure 5**). Similarly, in 2015 the tuber yield in treatments T1 to T5 was increased by 32–46% versus CK (**Figure 5**).

### Correlation of Fungal Abundance, Physical and Chemical Properties and Enzyme Activities

There were positive significant correlations between soil fungal abundance and soil water content (*r* = 0.661^∗∗^, *P* < 0.01), pH (*r* = 0.539^∗^, *P* < 0.05), and electrical conductivity (*r* = 0.571^∗^, *P* < 0.05); whereas, a negative significant correlation was found with available N content (*r* = -0.519^∗^, *P* < 0.05). In addition, a positive significant correlation was found between available N and alkaline phosphomonoesterase activity (*r* = 0.508^∗^, *P* < 0.05).

## Discussion

Changes in soil environment can affect composition, abundance, and activity of microbial communities (Cycon and Piotrowska-Seget, 2009). Plastic mulch has been reported to change soil environment, consequently the composition of microbial communities could be affected (Chen et al., 1998; Yu et al., 2015). It is established that soil temperature and moisture have a significant effect on soil microbial community, since they affect survival and activity of microbial species, including biocontrol microbes (De Curtis et al., 2012). The diversity of the soil fungi community, including *Fusarium*, can be affected by root-released compounds in continuous cropping field (Garbeva et al., 2004). In rhizosphere soils of peanut, eggplant, and potato, the number of bacteria and actinomycetes have been reported to decreased whereas that of fungi increased with extension in continuous cropping years (Allen et al., 1995; Li et al., 2012). The number and diversity of fungal dominant communities in rhizosphere soil is reported to increase because of potato continuous cropping (Meng et al., 2012). The results of this study showed that the fully mulching planting with ridge (T2) soil had the highest fungal diversity indices, and the half-mulching planting with furrow (T5) soil showed the lowest diversity indices. Moreover, there were significant differences among all six treated soils (*P* < 0.05). The diversity indices and variance analysis showed that fully-mulched managements (T2 and T3) increased soil fungal diversity in the rhizosphere soil of potato continuous cropping field compared to the traditional planting method (CK) and half-mulched managements (T4 and T5). Our results are consistent with the findings of Cao et al. (2012) and Qin et al. (2014) who reported that plastic film mulched enhanced soil nutrients and subsequently influenced soil microflora composition in corn field. This management practices might have increased soil moisture and other soil physicochemical properties and caused a change in the soil environmental properties leading to fungal growth, this proposition is supported by findings of (Zhang et al., 2006; Nyankanga et al., 2008). The higher fungi diversity in the T2 soils is a significant finding since beneficial fungi along with some bacteria perform important services related to water dynamics, nutrient cycling, and disease suppression (Lowenfels and Lewis, 2006). Along with bacteria, fungi are important as decomposers in the soil food web, converting hard to digest organic material into usable forms (Sylvia et al., 2005). Full mulching with plastic can improve soil moisture storage, rainfall use efficiency and water use efficiency, and increase soil temperature (Wang et al., 2011; Qin et al., 2014). Previous researches have reported that the number of soil fungi could increase with soil moisture (Bridge and Newsham, 2009; Zou et al., 2010).

Soil fungal diversity plays an important role in terrestrial ecosystems (Klironomos et al., 1997; Neher, 1999; Xu et al., 2008) because fungi can degrade soil organic matter (Jocrgensen and Wichern, 2008). Soil fungi are classified into soil habitants and soil invaders (Xing and Li, 2010). The former is a stable fungal class group completing their whole life cycle in soil, while the latter is a fungal group partly completing their life cycle in soil; Among soil fungi, there are plant pathogen such as some *Fusarium* (*F. coeruleum*, *F. sulphureum*, and *F. trichothecioides*), which can cause soil-borne diseases of potato, thus affecting potato yield and quality (Burgess and Summerel, 1992; Momma et al., 2010; Yu et al., 2015). We found that *Fusarium* was dominant fungus in potato continuous cropping soil but the abundance was decreased by half-mulched with ridge planting (T4) suggesting that this management may inhibit reproduction of *Fusarium*. Our result is consistent with the report by Li et al. (2013) who demonstrated that *Fusarium*, *Verticillium*, and *Rhizoctonia solani* were dominant in soils cropped to potato with the traditional management. As mentioned above, different mulching and ridge-furrow planting modes can cause different soil temperatures and soil water contents (Zhou et al., 2009; Qin et al., 2014). Soil fungi were more abundant and diverse in fully-mulched than in half-mulched planting management. Therefore, half-mulching planting management had a better preventive effect on reproduction of *Fusarium*. Plastic film mulching could change the biological characteristics of the soil and may have a negative impact on soil quality and sustainability. Qin et al., 2016 noted that fully-mulched planting pattern increased fungi population in potato continuous cropping field. Soil fungal populations are considered ‘xerophilous’ group with high moisture stress threshold values (Kouyeas, 1964; Qiu et al., 2014). Another research reported that fungi and actinomycetes are favored by drier soil conditions than bacteria (Stover, 1953). Several soil and crop management practices, such as fertilization (Ge et al., 2008; Yuan et al., 2013), crop rotation (Lupwayi et al., 1998), and tillage (Lupwayi et al., 2010) have been reported to produce a number of changes on soil microbial diversity with particular ecological importance. The results of the present study reveal important implications regarding the effects of the planting patterns on fungi diversity in a continuous cropping field. Increased in *Fusarium* species in the ridge-furrow and fully-mulched technique brought about a potential challenge in maintaining soil fertility, but this technology provides a potential opportunity of substantially increasing crop yields in semiarid rainfed regions. These results therefore, provide a clear understanding with respect to the response of soil fungi community to different planting patterns in a continuous potato cropping field, which is crucial to analyze the sustainability of the agricultural practice. The PCA of soil fungal community showed greater effect at early growth stage than the late growth stages. This results indicate the plant development stage also influenced fungi diversity significantly, a result which is inconsistent with the claim that the plant only has a minor influence on the constitution of the rhizosphere fungal community (Houlden et al., 2008). The reason for this inconsistency was likely that the different soil types and sampling methods lead to the different results.

Potato yield was greater in the fully-mulched with ridge planting with ridge cropping obtaining the highest under the continuous cropping condition. In contrast to our results, Liu and Siddique (2015) reported that RFM treatment did not increase potato yield at high altitude. The results difference could be attributed to the difference plastic material as black and white film is said to be suitable for potato and maize crops, respectively. In the present study, the complexity of soil–plant–microbial system could explain the large non-significant potato tuber among treatments with increased in continuous cropping years. Bakker et al. (2013) noted that potato competent microbes important for crop productivity is determined by site, cultivar and soil type that influence microbial community and consequently for better soil quality and crop yield.

## Conclusion

The ridge-furrow and fully-mulched technique increased fungi diversity in potato rhizosphere soil; soil fungal diversity indices were ranked as T2 > T3 > T1 > T4 > CK > T5. This result suggests that the fully-mulched planting method could promote reproduction of soil fungi. The fungi of the 6 soils were classified into 4 phyla, 20 classes, 48 orders, and 156 genera. Ascomycota was the dominant phylum, and Sordariomycetes was a dominant fungal class in all treatments; the dominant genus in all treatments was *Fusarium*, while the dominant genus was *Mortierella* in half-mulched with ridge planting (T4) soil. The lowest *Fusarium* occurred in T4 soil, indicating that the management could effectively inhibit reproduction of *Fusarium*. *Fusarium* species were also dominant in the fully-mulched treatments. The results also showed that mulching technology demonstrated increases in crop yields in semiarid rainfed regions. These results provide a clear understanding regarding the effects of planting patterns on fungi diversity and crop productivity in the semiarid regions of the world with a similar climate as that in northwestern China. However, several OTUs were unknown and future research may investigate these unknown fungi. In addition, further separation and identification of *Fusarium* will be required in future research.

## Author Contributions

SQ designed the work, XX and SQ collected the data and wrote the manuscript. SY, YL, and BY edited the manuscript and all authors approved the final version of this manuscript.

## Conflict of Interest Statement

The authors declare that the research was conducted in the absence of any commercial or financial relationships that could be construed as a potential conflict of interest..
